# Epidemiological and serological aspects of hepatitis A among children and teenagers in the city of Santos: a cross-sectional study

**DOI:** 10.1590/S1516-31802012000400005

**Published:** 2012-09-04

**Authors:** Maria Célia Cunha Ciaccia, Regina Célia Moreira, Alexandre Archanjo Ferraro, Marcílio Figueiredo Lemos, Isabel Takano Oba, Gilda Porta

**Affiliations:** I MD, Department of Pediatrics, Instituto da Criança (ICR), Faculdade de Medicina da Universidade de São Paulo (FMUSP), São Paulo, Brazil.; II MD, PhD. Scientific Researcher, Instituto Adolfo Lutz, São Paulo, Brazil.; III MD, PhD. Professor, Department of Pediatrics, Instituto da Criança (ICR), Hospital das Clínicas (HC), Faculdade de Medicina da Universidade de São Paulo (FMUSP), São Paulo, Brazil.; IV MD, PhD. Full Professor, Department of Pediatrics, Instituto da Criança (ICR), Hospital das Clínicas (HC), Faculdade de Medicina da Universidade de São Paulo (FMUSP), São Paulo, Brazil.

**Keywords:** Prevalence, Hepatitis A, Child, Serology, Epidemiology, Prevalência, Hepatite A, Criança, Sorologia, Epidemiologia

## Abstract

**CONTEXT AND OBJECTIVE:**

Viral hepatitis A is still a concern at public health level in Brazil and around the world, due both to the number of affected subjects and the possibility of complications in the acute forms. The Brazilian Ministry of Health estimates that at least 70% of this country’s population has already had contact with the hepatitis A virus (HAV). The aim here was to discover the prevalence of serological markers for the hepatitis A virus among children and teenagers at daycare facilities, kindergartens and elementary schools in the city of Santos.

**DESIGN AND SETTING:**

Cross-sectional study in kindergartens and elementary schools within the municipal education network in several regions of the city of Santos.

**METHOD:**

Students’ family members were surveyed using a questionnaire and 4,680 finger-prick blood samples were taken and assayed by means of the ELISA technique.

**RESULTS:**

The general prevalence of anti-HAV IgG was 9.72% and, of these cases, 74.6% were reactive to anti-HAV IgM. There was higher prevalence of anti-HAV IgG among older children, females, children who played in streams, those whose homes were not connected to the sewage system, those whose parents had low education levels, those with low household income and those who did not live along the seashore. The prevalence of anti-HAV IgM peaked in the early years and subsequently fell, and it was lower on the hills and in the Northwestern Zone.

**CONCLUSION:**

The general prevalence of serological markers for hepatitis A was low in Santos.

## RESUMO

**CONTEXTO E OBJETIVO:**

A hepatite viral A continua sendo uma preocupação em nível de saúde pública no Brasil e no mundo, tanto pelo número de indivíduos atingidos, como pela possibilidade de complicação das formas agudas. O Ministério da Saúde estima que pelo menos 70% da população do Brasil já tiveram contato com o vírus da hepatite A. O objetivo foi conhecer a prevalência de marcadores sorológicos do vírus da hepatite A em crianças e adolescentes de creches e escolas de ensino infantil e fundamental na cidade de Santos.

**TIPO DE ESTUDO E LOCAL:**

Estudo transversal em pré-escolas e de ensino fundamental da rede municipal em diversas regiões da cidade de Santos.

**MÉTODO:**

Foi aplicado um questionário aos familiares dos estudantes e coletadas 4.680 amostras de sangue através de punção capilar para realização da sorologia pela técnica ELISA.

**RESULTADOS:**

A prevalência geral do anti-HVA IgG foi de 9,72% e, desses, 74,6% foram anti-HVA IgM reagentes. A prevalência de anti-HVA IgG foi maior entre as crianças mais velhas, meninas, aquelas que brincavam em córregos, sem rede de coleta de esgoto em sua moradia, de pais com baixa instrução, de baixa renda familiar e aquelas que não eram moradoras da orla. A prevalência de anti-HVA IgM teve pico nos primeiros anos e posterior queda e, no morro e Zona Noroeste, foi mais baixa.

**CONCLUSÃO:**

A prevalência geral dos marcadores sorológicos para hepatite A foi baixa em Santos.

## INTRODUCTION

Viral hepatitis A is still a concern at public health level in Brazil and around the world, due both to the number of affected subjects and the possibility of complications in the acute forms. The Brazilian Ministry of Health estimates that at least 70% of this country’s population has already had contact with the hepatitis A virus (HAV).^1^

It is known that sanitary and hygiene conditions influence hepatitis A incidence, since the major virus transmission route is fecal-oral. Studies have shown greater hepatitis A prevalence with increasing age,^2^ and also with poor socioeconomic and sanitary conditions.^2^^,^^3^ In Brazil, Clemens et al.^4^ evaluated the prevalence of anti-HAV among 3653 subjects aged 1 to 40 years, in four Brazilian state capitals: in the Northern region, it was 92.8%; in the Northeastern region, 76.5%; and in the Southern and Southeastern regions, 55.7%.

The severity of the clinical picture is directly related to the patient’s age. About 1% of hepatitis A cases can evolve into the fulminant form.^5^^,^^6^ Despite improvements in the socioeconomic conditions of populations, hepatitis A epidemics and outbreaks continue to occur,^7^ even in developed countries,^8^ and they are a major public health problem. Considering that young adults are more susceptible to the infection and that disease severity for this age range is high, healthcare professionals need to be aware of the prevalence of viral hepatitis A in order to identify, evaluate and control epidemics and calculate the impact of vaccination programs.^9^^,^^10^^,^^11^^,^^12^ It is worth emphasizing that some studies have showed that vaccination against hepatitis A is capable of preventing disease dissemination during an outbreak, and also of protecting patients’ household contacts.^13^

In Brazil, there have been few prevalence studies on population samples representative of hepatitis A. It is therefore difficult to generalize the findings or make comparisons between these studies.

Therefore, we proposed to identify the knowledge of the epidemiological and serological aspects of hepatitis A among children and teenagers enrolled at daycare facilities, kindergartens and elementary schools within the municipal education network of the city of Santos, Brazil. We believe that this is important for enabling actions and policy decisions directed towards the healthcare sector.

## OBJECTIVE

The aim of this study was to assess the prevalence of immunoglobulin G (IgG) and immunoglobulin M (IgM) anti-hepatitis A virus (anti-HAV) antibodies, thus revealing any cases of subclinical acute infection by the hepatitis A virus among children and teenagers enrolled in the municipal education network of the city of Santos, according to age range and socioeconomic level.

## METHODS

Santos is the largest city on the coast of the state of São Paulo, with an area of 280,300 km^2^ and a population of nearly half a million. Its medical care resources are centered around the public healthcare system (Sistema Único de Saúde, SUS) and service agreements. There have not been any really representative evaluations on the prevalence of antibodies against hepatitis A among children and teenagers in Santos.

A cross-sectional study was conducted between June 28 and December 14, 2007, in which 4,680 finger-prick blood samples were collected from children and teenagers enrolled at daycare facilities, kindergartens and elementary schools within the municipal education network in several regions of the city of Santos. At the same time, a survey questionnaire was applied to members of the children and teenagers’ families. The following were taken to be exclusion criteria: students who were absent after three requests to take part in the study, those who did not bring the vaccination record card (n = 374) and those who had received the vaccine against hepatitis A (n = 57) for analysis of the anti-HAV IgG serological markers.

This project was approved by the Ethics Committee of Hospital das Clínicas (HC) and of Faculdade de Medicina da Universidade de São Paulo (FMUSP), by the Ethics Committee of the Health Department of Santos and by the Education Department of Santos. An informed consent statement was presented to the persons responsible for the children and teenagers and, after they had approved it by signing it, the questionnaire was applied and collection of finger-prick blood samples was started.

### Operationalization

The first part of the study consisted of randomizing the students and telephoning their parents or the persons responsible for them, in order to apply a questionnaire seeking information regarding student identification, age, gender, birth date, parents’ education level and parents’ profession, household income, individual and family history, physical environment and vaccination record card. The second part consisted of carrying out the sample collection and the third part consisted of laboratorial testing.

### Sample calculation

This study was part of a population survey about the prevalence of hepatitis A, B and C. To determine the sample size, an expected frequency of 1% (the expected frequency of hepatitis B would be less than this), acceptable error of 0.5% and confidence level of 95% were calculated using the Epi Info software, version 6. The total population was 29,589 students enrolled within the municipal education network of Santos: 2,050 at daycare facilities, 6,079 in kindergartens, 16,026 in elementary school from 1^st^ to 4^th^ grade and 5,434 teenagers from 5^th^ to 8^th^ grade. From this population, the total sample achieved was 4,680 students, i.e. 880, 1,220, 1,390 and 1,190, respectively for each education stratum. The sample was then divided into equally sized groups in each of five regions of the City of Santos. The regions for this study were chosen based on the socioeconomic differences that exist among them.

In each of these regions, two daycare facilities were randomized (except for the hill region, where three daycare facilities were randomized, since the sufficient number was not reached with only two), as well as two kindergarten education units, two elementary schools from 1^st^ to 4^th^ grade and two from 5^th^ to 8^th^ grade. In each school unit, the classes and the number of students in each of them were randomized, always in equal sizes, until the total number for the sample was reached.

### Sample collection

The blood collection consisted of finger puncture using an appropriate disposable lancet, on a filter paper containing two circles of 2.5 centimeters in diameter. After collection, the filter paper with the student’s identification remained for two hours in a container for drying and was then stored at - 20 °C in closed small transparent plastic bags. The serological tests performed were IgG anti-HAV and IgM anti-HAV (the latter was performed when IgG was positive), by means of the enzyme immunoassay. The tests were carried out in the Hepatitis Laboratory of Institute Adolfo Lutz, in São Paulo.

### Data processing and analysis

The analysis was done with the aid of the Stata software, version 9.0. To compare means, we used the Mantel-Haenszel chi-square and the chi-square trend for ordinal categories. For risk analysis, we calculated the prevalence ratio according to category, and its 95% confidence interval, using Poisson regression. To ascertain the effect of independent variables, multivariate Poisson regression was used, with the calculation of the linear trend or heterogeneity in cases in which there was more than one risk category. The contributors in this analysis were 3928 individuals whose data were complete. The value for rejecting the null hypothesis was set at P < 0.05.

## RESULTS

The ages of the study population ranged from seven months to 18 years and one month, and the mean age was eight years and four months. The general prevalence of reactive anti-HAV IgG serological markers was 9.72% and, among these individuals, 74.7% were also found to be reactive to anti-HAV IgM, as shown in Table 1.

**Table 1.** General prevalence of the anti-HAV IgG and anti-HAV IgM serological markers when anti-HAV IgG was reactive

	n ( % )	
	Anti-HAV IgG	Anti-HAV IgM
Non-reactive	3,836 (90.3)	105 (25.4)
Reactive	413 (9.7)	308 (74.6)
Total	4,249 (100.0)	413 (100.0)

In the cases that were reactive to the anti-HAV IgG and IgM serological markers, the prevalence of evident symptoms or knowledge of ever having had hepatitis, on the part of the student or his/her family, was low. Most of them said that they had never had any symptoms and were unaware of ever having had hepatitis (95.6%). Among the symptoms presented, jaundice was the most frequent (3.9%).

Table 2 shows the prevalence of anti-HAV IgG and IgM among the population. All the variables were statistically associated with presence of anti-HAV IgG, with higher prevalence among older children, females, children who were in the habit of playing in streams, those whose homes were not connected to the sewage system, those whose parents had low education levels, those with low family income and those who did not live along the seashore (no differences between the other areas).

**Table 2.** Prevalence of reactive serological findings for anti-HAV IgG and anti-HAV IgM (subsample of individuals who were IgG-positive), according to the characteristics of the population

Variables	Anti-HAV IgG (N = 4,249)			P^*^	anti-HAV IgM (N = 413)			P^*^
	N	n	%		N	n	%	
Education groups								
Daycare facility	835	21	2.5	< 0.001^†^	21	16	76.2	< 0.001^†^
Kindergarten	1,188	65	5.5		65	60	92.3	
Elementary 1^st^ - 4^th^	1,263	192	15.2		192	167	87.0	
Elementary 5^th^-8^th^	963	135	14.0		135	63	46.7	
Gender								
Male	2,119	186	8.8	0.039	186	137	73.7	0.855
Female	2,130	227	10.7		227	169	74.5	
Playing in streams								
No	3,990	358	9.0	< 0.001	358	265	74.0	0.934
Yes	258	55	21.3		55	41	74.6	
Home connected to sewage system								
Yes	3,812	341	9.0	< 0.001	341	252	73.9	0.847
No	434	72	16.6		72	54	75	
Father’s education level								
Illiterate	106	24	22.6	< 0.001^†^	24	15	62.5	0.135^†^
Incomplete elementary	1,514	233	15.4		233	168	72.1	
Completed elementary	673	57	8.5		57	45	79.0	
Incomplete high school	390	16	4.1		16	14	87.5	
Completed high school	1,050	38	3.6		38	28	73.7	
University-level education	221	7	3.8		7	5	71.4	
Mother’s education level								
Illiterate	97	24	24.7	< 0.001^†^	*24*	15	62.5	0.920^†^
Incomplete elementary	1,479	238	16.1		238	179	75.2	
Completed elementary	626	52	8.3		52	39	75.0	
Incomplete high school	469	27	5.8		27	24	88.9	
Completed high school	1,346	55	4.1		55	37	67.3	
University-level education	197	8	4.1		8	4	50.0	
Family income								
1-2 minimum wages	2,320	301	13.0	< 0.001^†^	301	225	74.8	0.558^†^
3-4 minimum wages	1431	95	6.6		95	70	73.7	
5 + minimum wages	495	17	3.4		17	11	64.7	
Areas								
Seashore	839	49	5.8	0.001	49	36	73.5	< 0.001
Center	876	95	10.8		95	83	87.4	
Intermediate	854	90	10.5		90	74	82.2	
Hills	769	80	10.4		80	55	68.8	
Northwestern Zone	911	99	10.9		99	58	58.6	

N = total sample; n = sample with positive serological test; % = prevalence of positivity in the serological testing; the incongruence of some results from addition is due to ‘missing data’; ^*^Chi-square; ^†^Chi-square for trend.

The prevalence of anti-HAV IgM, as shown in Table 2, did not differ between the categories, except for the age (peak in the early years and subsequent fall) and the area of the city (lower on the hills and in the Northwestern Zone).

Table 3 shows the risk associated with each category of the variable studied for IgG antibody positivity. The greatest risks were associated with parental education (prevalence reason, PR = 7.15 with 95% confidence interval, CI, of 3.08-16.59 for the father to be illiterate; and PR = 6.09 with 95% CI of 2.74-13.56 for the mother to be illiterate), and the lowest risk was associated with family income (PR = 0.26 with 95% CI of 0.16-0.43 for the family to be wealthy).

**Table 3.** Uni and multivariate analysis on the risk of positive findings of anti-HAV IgG relating to presence of characteristics of the population (n = 3,928)

Variable	Univariate analysis			Multivariate analysis (variables reciprocally adjusted)		
	PR	95% CI	P	PR	95% CI	P
Groups educations						
Daycare facility	1.00		< 0.001^*^	1.00		< 0.001^*^
Kindergarten	2.18	1.33-3.56		2.21	1.28-3.80	
Elementary 1^st^-4^th^	6.04	3.85-9.48		5.81	3.52-9.61	
Elementary 5^th^-8^th^	5.57	3.52-8.83		6.11	3.67-10.19	
Gender						
Male	1.00		0.050	1.00		0.373
Female	1.21	1.00-1.47		1.10	0.89-1.35	
Playing in streams						
No	1.00		< 0.001	1.00		0.006
Yes	2.38	1.79-3.16		1.54	1.13-2.10	
Home connected to sewage system						
Yes	1.00		< 0.001	1.00		0.041
No	1.85	1.44-2.39		1.36	1.01-1.84	
Father’s education level						
Illiterate	7.15	3.08-16.59	< 0.001^*^	3.02	1.21-7.48	< 0.001^*^
Incomplete elementary	4.86	2.29-10.30		2.35	1.05-5.26	
Completed elementary	2.67	1.22-5.86		1.79	0.78-4.11	
Incomplete high school	1.30	0.53-3.15		1.05	0.42-2.66	
Completed high school	1.14	0.51-2.56		1.11	0.48-2.53	
University-level education	1.00			1.00		
Mother’s education level						
Illiterate	6.09	2.74-13.56	< 0.001^*^	1.80	0.74-4.38	< 0.001^*^
Incomplete elementary	3.96	1.96-8.02		1.32	0.61-2.85	
Completed elementary	2.05	0.97-4.31		0.87	0.39-1.94	
Incomplete high school	1.42	0.64-3.12		0.80	0.34-1.85	
Completed high school	1.01	0.48-2.11		0.67	0.31-1.45	
University-level education	1.00			1.00		
Family income						
1-2 minimum wages	1.00		< 0.001^*^	1.00		0.001^*^
3-4 minimum wages	0.51	0.41-0.64		0.69	0.54-0.90	
5 + minimum wages	0.26	0.16-0.43		0.52	0.31-0.88	
Areas						
Seashore	1.00		0.003^†^	1.00		0.513^†^
Center	1.86	1.31-2.62		1.35	0.94-1.94	
Intermediate	1.80	1.27-2.56		1.33	0.92-1.93	
Hills	1.78	1.25-2.54		1.19	0.82-1.73	
Northwestern Zone	1.86	1.32-2.62		1.27	0.88-1.84	

PR = prevalence ratio; 95% CI = 95% confidence interval; ^*^testing of linear trends; ^†^testing of heterogeneity.

Multivariate analysis on anti-HAV IgG positivity showed that the variables were all independent of each other, except for gender and the area of the city, and were not statistically significant. The greatest risk was among the teenagers: 6.11 times higher than among the children in daycare.

## DISCUSSION

The sampling of this study was probabilistic, thus assuring its representativeness, and the proportions were maintained when age ranges according to the census data were used.

The use of capillary blood collected on a filter paper for serological testing facilitates the collection, transportation and storage of the samples, and the results reported in the literature already show that such testing is valid for hepatitis A investigations.^14^ Thus, blood collection by means of a lancet on filter paper for determining antibody levels provides an excellent opportunity to investigate diseases in representative populations.

The presence of anti-HAV IgG indicates previous exposure to the virus or vaccination. Determination of its prevalence enables evaluation of the immunological condition of such populations of children and teenagers without vaccination against HAV, thus indicating natural infection. The general prevalence of serological markers for hepatitis A in our study (9.7%) was lower than in other regions of Brazil, with reports ranging from 32.3% in the Central-Western region,^15^ in 2008, to 60% among children under the age of 10 years in Lábrea, in the Brazilian Western Amazon region,^16^ in 2009. Regarding worldwide epidemiology, variations have been found, depending on the location.^17^^,^^18^ It was found to be rare in Japan,^18^ among subjects under the age of 44 years, and reached up to 80% in Turkey,^19^ among children under the age of nine years.

We found that anti-HAV IgG was more prevalent among females, which had not been reported in the majority of the data in the literature. In the semi-arid region of the state of Bahia,^20^ and in the Brazilian Western Amazon region^20^ among children between 6 months and 14 years of age, the prevalence of these markers was found to be similar between the genders.

The strong relationship between positivity of anti-HAV IgG and socioeconomic conditions found in the present study is concordant with the data in the literature. Pinho et al.^3^ showed that the prevalence of anti-HAV IgG in Campinas was 95% in the group of low socioeconomic level and 19.6% in the group of high socioeconomic level. Ferreira et al.^2^ evaluated the relationship between the prevalence of hepatitis A and socioeconomic level in Porto Alegre, and found that the prevalence was 51% in the low-level group, and 11% in the high-level group. In Vila Velha, Espírito Santo,^21^ there was a correlation between positive serological tests for hepatitis A and lower access to filtered water, lower access to the sewage system, habits of swimming in rivers or the sea and a greater number of people living in the same room. In the Brazilian Western Amazon region,^16^ it was also associated with a greater number of people in the family. In Greece,^22^ a strong relationship was shown between positive serological tests for hepatitis A and a low level of education among the parents, a greater number of people per room and lower access to water, among children with an average age of nine years.

In our series, we observed that a large number of teenagers were at high risk of infection, because even with the gradual increase in prevalence of anti-HAV IgG antibodies, it remained low. Clemens et al.^4^ showed that the prevalence of anti-HAV IgG antibodies increased with age, in four Brazilian regions.

We observed that there had been an outbreak of hepatitis A virus, by analyzing the positivity of anti-HAV IgM. We found in this study that there was no influence from the presence or absence of a connection to the sewage system among these children’s homes, or from the habit of playing in streams near their homes.

Most of the children who were reactive to anti-HAV IgM did not show any symptoms, and these data are in agreement with the literature. No liver enzyme tests were performed in most of these children with the presence of anti-HAV IgM, because they were asymptomatic. Vitral et al.^23^ demonstrated in a review of the literature covering 1980 to 2002, in Brazil, that infection by the hepatitis A virus is very often asymptomatic or develops without jaundice in children, and that the symptoms increase with age.

The high proportion of asymptomatic cases in lower age ranges probably makes it difficult to estimate the magnitude of the events and to decide on timely intervention measures that would prevent diffusion of epidemics. In this manner, the infection reaches older age groups. Continuing presence of susceptibility to hepatitis A virus among adults indicates that although the socio-environmental conditions may have improved, cases of acute disease at adult ages can still occur, with the possibility of greater severity. At the same time, since there are younger children susceptible to the virus, studies on vaccination strategies can be designed, given that this is a common disease that is preventable through immunization.

Although our data confirm that a change in the epidemiological profile of hepatitis A has taken place, the current preventive measures regarding sanitation, education level and housing still remain deficient in Santos, the coastal city with the largest port in Brazil.

Based on these epidemiological data, we call on government spheres to make a response directed towards dealing with this major public health problem. Despite socioeconomic improvements, a number of environmental factors have remained unchanged over the years, such as poor conditions of hygiene and family education; unsuitable sanitary conditions; inadequate sewage system provision by the local administration; and the authorities’ inability to resolve the lack of sewage network in such a way that transmission of infections can be prevented: not only hepatitis A, but also other enteric infections.

## CONCLUSIONS

It is concluded in this study that the general prevalence of serological markers for hepatitis A was low, compared with the literature.
